# SMART: Structured Missingness Analysis and Reconstruction Technique for credit scoring

**DOI:** 10.1038/s41598-025-99997-4

**Published:** 2025-04-29

**Authors:** Seongil Han, Haemin Jung, Paul D. Yoo

**Affiliations:** 1 Department of Computer Science, University of Suwon, Hwaseong, South Korea; 2 Department of Industrial and Management Engineering, Korea National University of Transportation, Chungju, South Korea; 3 School of Computing and Mathematical Sciences, University of London, Birkbeck College, London, UK

**Keywords:** Generative adversarial imputation networks, Randomized singular value decomposition, Imputation, Missing values, Credit scoring, Computer science, Information technology

## Abstract

The Basel Accord emphasizes the necessity of employing internal data models to manage key credit risk components, including Probability of Default (PD), Loss Given Default (LGD), and Exposure At Default (EAD). Among these, internal datasets are critical for estimating PD, a fundamental measure of borrower creditworthiness. Nevertheless, practical application often faces challenges due to incomplete datasets, which can skew analyses and undermine the accuracy of credit scoring models. Traditional approaches to addressing missing data, such as sample deletion or mean imputation, are widely used; however, they often prove insufficient for accurate prediction. Consequently, imputation methods are typically favored over deletion, as they allow for the full utilization of available data. Recent advancements have introduced more sophisticated techniques, such as Generative Adversarial Imputation Networks (GAIN), which utilize a generative adversarial network to model data distributions and impute missing values with greater precision than conventional methods. Building on these developments, this study proposes a novel imputation approach, SMART (Structured Missingness Analysis and Reconstruction Technique) for credit scoring datasets. SMART consists of two primary stages: first, it normalizes and denoises the dataset using randomized Singular Value Decomposition (rSVD), followed by the implementation of GAIN to impute missing values. Experimental results demonstrate that SMART significantly outperforms existing state-of-the-art methods, particularly in high missing data contexts (20%, 50%, and 80%), with improvements in imputation accuracy of 7.04%, 6.34%, and 13.38%, respectively. In conclusion, SMART represents a substantial advancement in handling incomplete credit scoring datasets, leading to more precise PD estimation and enhancing the robustness of credit risk management models.

## Introduction

Credit scoring can be modeled using records or datasets that include various input features, such as demographic information, assets, income, and payment behavior, which are relevant to assessing creditworthiness. However, real-world datasets frequently exhibit missing values or incomplete features due to several reasons, including unrecorded observations or corrupted data^1–3^. For example, the UCI Machine Learning Repository, a widely utilized dataset collection, reveals that 45% of its datasets contain missing values^4^. Incomplete data are also a common occurrence in observational studies across various fields, such as social sciences and clinical trials^5–7^. The presence of missing values diminishes the number of available samples for analysis and may distort the analysis when classification models are employed for prediction^8,9^. Consequently, this limitation adversely impacts model performance, as missing values or incomplete datasets can result in misclassification in the context of creditworthiness assessment^10^. Furthermore, machine learning models typically require complete data for training prior to the prediction process^11,12^. Given the necessity for dataset completeness in statistical and machine learning models, it is imperative to address missing values appropriately^13^.

Nonetheless, this issue has often been overlooked, and simplistic methods such as excluding samples with missing values or employing mean imputation have been commonly utilized to manage incomplete datasets in credit scoring analyses^9,14^. The primary goal of addressing missing values is to estimate values that closely resemble those of the complete underlying dataset^13^. These approaches can be classified into two categories: deletion and imputation^15^.

Since deletion methods reduce the sample size by excluding instances with missing values, they can lead to distorted analyses and are generally sub-optimal. If a substantial portion of the samples is incomplete, this reduction in sample size can result in significant errors^6^. Conversely, imputation methods, which involve replacing missing data with substituted values, are frequently employed. This process is referred to as missing data imputation^16^. Imputation is widely regarded as the most appropriate and valid approach for handling incomplete datasets^17^. Imputation methods can be classified into statistical and Machine Learning (ML) approaches, or generative methods, based on the characteristics of the imputation process^18^.

Therefore, the capability to manage missing values or incomplete datasets is essential for classification tasks to mitigate significant errors or incorrect estimations. Numerous studies, grounded in statistical and machine learning methodologies, have been conducted to address this issue, revealing that no single method consistently guarantees efficiency. This suggests that the effectiveness of these methods is dependent on the specific context of the problem, including factors such as the number of samples, the number of features, the pattern of missing data, and the percentage of missing data within the dataset^9^.

This study aims to extend the GAIN-based imputation technique by proposing a novel imputation method named SMART (Structured Missingness Analysis and Reconstruction Technique) to address the issues of missing values in credit scoring datasets for classification purposes. Initially, missing values are generated within the complete dataset. Subsequently, these missing values are imputed using various statistical and machine learning methods to evaluate the impact of different imputation techniques on the performance of credit scoring models. In particular, machine learning-based imputation methods such as Multiple Imputation by Chained Equations (MICE)^19^, MissForest^20^, Generative Adversarial Imputation Networks (GAIN)^1^, and variants of GAIN will be comparatively analyzed within the context of credit scoring. The study will examine whether these imputation techniques for handling missing values can enhance classification performance in credit scoring. The primary objective of this study is to assess the robustness and effectiveness of SMART, as well as to compare its imputation performance with that of GAIN, its variants, MissForest, and MICE.

The key contributions of this study include:

- To illustrate the efficacy of the denoising method employing randomized Singular Value Decomposition (rSVD) within credit scoring datasets
- To present advancements in GAIN imputation when combined with rSVD, demonstrating enhanced imputation performance in incomplete credit scoring datasets, and to compare this performance with benchmarks including the original GAIN, its variants, and conventional statistical and machine learning imputation techniques
- To propose an architecture for a credit scoring model capable of effectively handling datasets with missing values
- To establish new benchmark results that exceed the performance of the state-of-the-art model by^1^ on the Default of Credit Card Clients (DC) dataset for missing value imputation

We hypothesize that the proposed SMART will not only mitigate noise in redundant and noisy datasets but also develop models proficient in performing accurate imputation for incomplete credit scoring datasets.

The remainder of this paper is structured as follows: “Related work” reviews related studies and identifies the gaps in current technologies. “Methods” details the proposed SMART and its novel concepts. “Results” presents the results, evaluating the performance of SMART against benchmarks from recent studies. “Discussion” discusses the key findings, limitations, and potential directions for future work. Finally, “Conclusion” concludes with a summary of the study’s findings.

## Related work

This section reviews related studies on imputation methods and mechanisms for addressing missing values.

### Imputation methods for missing values

Imputation methods for addressing missing values can be broadly classified into two categories: simple imputation methods and multiple imputation methods.

Single imputation methods, such as mean, median, mode, and regression, involve replacing missing values with a single estimated value. Among these, mean imputation^21^, which substitutes missing values with the overall sample mean, is widely and easily used^22^. However, this approach can underestimate standard error and variance and distort the underlying data distribution by ignoring the relationships between features^23^. Regression imputation, which employs existing (complete) data in features through a regression model to predict and substitute missing values, is considered an improvement over mean imputation. Nonetheless, it also encounters similar issues regarding standard error and variance^24^. Consequently, the limitations of single imputation methods hinder their ability to achieve an acceptable level of performance^25^.

Multiple imputation methods, such as Expectation Maximisation (EM)^26^ and Multiple Imputation by Chained Equations (MICE)^19^, replace missing values with a set of values, thus addressing the limitations of simple imputation methods^27^. In contrast to single imputation, multiple imputation generates several values based on a predictive distribution. These values are then combined into a single value after the analysis process to impute the missing data. Although multiple imputation mitigates the unreliability of single imputation methods^28^, it is computationally intensive due to the difficulty in determining the optimal number of iterations for convergence^15^. Furthermore, MICE, which utilizes regression-based methods such as linear regression, exhibits limited performance in capturing non-linearity^28,29^. Consequently, this limitation may hinder the accurate reflection of interactions between features during imputation.

In contrast, machine learning methods such as K-Nearest Neighbors (KNN)^30^ and MissForest^20^ are frequently employed for imputing missing values, as these methods do not rely on assumptions regarding data distribution^9^. MissForest, which utilizes Random Forests (RF), has been shown to effectively handle non-linearity by providing estimates for missing values based on the dataset^20^. Research indicates that MissForest typically performs better than MICE in terms of imputation accuracy^31^.

Moreover, Generative Adversarial Imputation Networks (GAIN)^1^, which utilize a GAN-based approach for substitution, have been introduced and shown to yield promising results in imputing missing values in tabular datasets. Yoon et al.^1^ proposed the use of a generative model for estimating missing values through a GAN architecture in an adversarial manner. As highlighted, GANs are recognized for their accuracy and effectiveness in capturing latent patterns within complex and non-linear datasets, owing to their ability to model the underlying distribution of the original data^25^. Furthermore, the research conducted by Yoon et al.^1^ demonstrated that GAIN exhibits greater robustness compared to existing imputation methods, such as AutoEncoder and MissForest. Table 1 provides examples of various imputation methods.

**Table 1:** The examples of imputation methods.

Method	Category	Reference
Mean imputation	Mean	^21^
Expectation maximisation (EM)	EM	^26^
EM with bootstrapping	EM	^32^
K-Nearest Neighbour (KNN)	KNN	^30^
MICE	ML-based regression trees	^19^
MissForest	ML-based random forest	^20^
GAIN	GAN-based neural networks	^1^

Nazabal et al.^33^ introduced the HI-VAE model, which is designed to estimate Heterogeneous Incomplete (HI) tabular data comprising both continuous and discrete values, utilizing a Variational AutoEncoder (VAE). Their findings indicated that HI-VAE provides improved predictive performance on complete data compared to the original incomplete data. Camino et al.^34^ proposed enhancements to the imputation performance of GAIN and VAE, demonstrating that their methods achieve superior results on real-world datasets. Building upon advancements in Generative Adversarial Network (GAN) methodologies, Friedjungová et al.^35^ introduced the Wasserstein GAIN (WGAIN) framework to improve the stability and performance of the original GAIN imputation approach. This study systematically evaluates the effectiveness of both generative and non-generative models for feature reconstruction in the presence of missing data. These methods are compared against established imputation techniques, including KNN and MICE. The experimental findings demonstrate that WGAIN consistently outperforms GAIN, KNN, and MICE, exhibiting superior imputation accuracy across various benchmark settings.

Furthermore, Awan et al.^25^ proposed a variant of the GAIN algorithm, termed CGAIN, which utilizes Conditional GAN (CGAN) to generate synthetic values for missing data. Their study provided a comparative analysis of CGAIN’s performance against existing imputation methods. Neves et al.^36^ introduced three GAN-based imputation techniques: Slim GAIN (SGAIN), a variant of the original GAIN model, and two extensions of the WGAIN framework-Wasserstein Slim GAIN with Clipping Penalty (WSGAIN-CP) and Wasserstein Slim GAIN with Gradient Penalty (WSGAIN-GP), both of which serve as variants of WGAIN-and evaluated their performance across a range of missing data rates. Their empirical findings demonstrate that, with GAIN as the benchmark, these methods consistently achieved superior results in terms of imputation quality, computational efficiency, and predictive performance in downstream tasks such as classification.

### Mechanism for missing values

Since the approach to handling missing values is heavily influenced by the underlying mechanism of their occurrence, it is crucial to analyze and account for the nature and impact of missingness within credit scoring datasets^17^.

**Figure 1:**
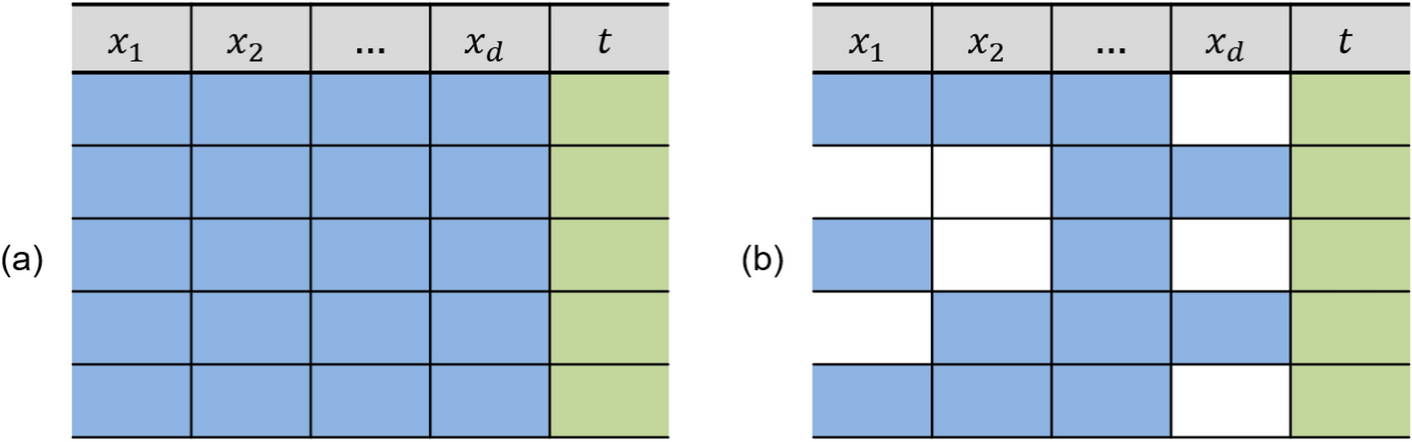
(**a**) The complete data without missing values on the features and (**b**) incomplete data with missing values on the features, where $x_{1}, x_{2}, \ldots , x_{d}$ represent the features, and *t* denotes the label.

A dataset without missing values can be denoted as *X*, while a dataset with missing values is denoted as $X_{M}$. Consequently, $X_{M}$ consists of two components:

1 $$\begin{aligned} X_{M} = \{X_{o}, X_{m}\}, \end{aligned}$$

where $X_{o}$ denotes the observed and complete subset of the dataset, while $X_{m}$ represents the missing and incomplete subset. The matrix *M*, known as the missing data indicator matrix, is used to denote the presence or absence of data. Specifically, *M* is a binary matrix that indicates which elements of $X_{M}$ are observed and complete and which are missing or incomplete^4^. Figure 1 provides a schematic representation of the data structure, contrasting (a) the complete dataset with no missing values and (b) the incomplete dataset with missing entries across feature dimensions. The figure visually highlights the presence and distribution of missingness, where $x_{1}, x_{2}, \ldots , x_{d}$ denote the feature variables and *t* represents the corresponding label.

Since missing values $X_{m}$ can be estimated from the observed values $X_{o}$ through various imputation methods, missing data can be classified into three types based on the mechanism of their missingness. This classification pertains to how and why the data components are absent^37^. The characteristics of missingness are typically categorized as follows^4,36,38^:

1. Missing completely at random (MCAR)
2. Missing at random (MAR)
3. Missing not at random (MNAR)

Missing Completely At Random (MCAR) arises when the probability *P* of a data point being missing is independent of both the observed data and the missing data itself. This condition reflects a high degree of randomness, where the likelihood *P* of data or features being absent is not influenced by any characteristics of the data^4^.

2 $$\begin{aligned} P(M|X_{m}) = P(M) \end{aligned}$$

Missing At Random (MAR) occurs when the probability of missing data depends solely on the observed data and not on the missing data itself. This situation represents a moderate level of randomness, where the likelihood of data being missing is influenced by the observed values but remains independent of the values that are missing^4^.

3 $$\begin{aligned} P(M|X_{m}) = P(M|X_{o}) \end{aligned}$$

Missing Not At Random (MNAR) occurs when the probability *P* of a data point being missing is dependent on both the observed data and the missing data itself. This condition reflects a low level of randomness, where the likelihood *P* of data or features being absent is influenced by both the observed values and the values that are missing^4^.

4 $$\begin{aligned} P(M|X_{m}) = P(M|X_{o}, X_{m}) \end{aligned}$$

The classification of missingness characteristics is vital, as different imputation methods are applicable based on specific assumptions or conditions. When data are categorized as Missing Completely At Random (MCAR) or Missing At Random (MAR), the missing data mechanism is considered ignorable^4^. This means that, for data exhibiting MCAR or MAR, imputation methods can be applied without regard to the underlying cause of the missingness, and it is feasible to estimate the missing values^7^. As a result, a significant portion of research on missing data imputation has been concentrated on scenarios where the missingness is either MCAR or MAR^4,36,39^.

This study confines its scope to the Missing Completely At Random (MCAR) mechanism, guided by several justifiable considerations. First, modeling more complex missingness mechanisms such as Missing At Random (MAR) or Missing Not At Random (MNAR) often necessitates substantial domain-specific knowledge and nuanced understanding of the data’s generating process-requirements that are typically infeasible when working with third-party datasets. In contrast, the MCAR assumption posits that the likelihood of missingness is independent of both observed and unobserved data, thereby simplifying the analytical framework. Second, MCAR permits the use of less complex machine learning models, as it eliminates the need to explicitly model inter-feature dependencies. Third, a large body of literature on contemporary imputation methods-spanning both discriminative and generative approaches-implicitly or explicitly assumes an MCAR setting. Lastly, the adoption of a uniform missingness mechanism ensures methodological consistency and facilitates equitable comparisons against existing benchmarks.

## Methods

### The framework of SMART

The Structured Missingness Analysis and Reconstruction Technique (SMART) methodology comprises four sequential stages:

1. Collecting Default of Credit card clients (DC) dataset
2. Rescaling and denoising the original dataset using randomized Singular Value Decomposition (rSVD)
3. Imputing missing values in the incomplete dataset using the Generative Adversarial Imputation Network (GAIN)
4. Performing classification on the dataset post-imputation

These stages must be executed sequentially to achieve the desired level of effectiveness.

The Default of Credit card clients (DC) dataset^40^ encompasses various attributes including demographic details, payment history, default payment status, and records of delinquency for credit card clients. Originating from prior research in credit scoring, this dataset is also accessible through the UCI Machine Learning Repository. It consists of 30,000 instances, categorized into 23,364 samples of good credit and 6,636 samples of bad credit. In this dataset, a bad credit instance is identified by the target variable ‘default.payment.next.month’ being equal to 1, which signifies that the client has defaulted on payment. Conversely, a good credit instance is characterized by the target variable being equal to 0, indicating that the client has not defaulted. The dataset is a widely used, publicly available benchmark for credit risk modeling. Notably, it is originally complete and devoid of missing values, making it particularly suitable for controlled evaluations of imputation methods under simulated missingness. This dataset has been employed in several prior studies^1,25,34,36,41^, including the original GAIN paper, thereby enabling meaningful and reproducible comparisons. Its consistent use in the literature provides a strong foundation for assessing SMART’s performance relative to established baselines on a well-recognized benchmark.

The initial dataset reveals that the minority class constitutes 22.12% of the total, with an Imbalance Ratio (IR) of 3.52, calculated as the ratio of the majority class to the minority class. The dataset comprises 23 features, not including the target variable, which can be directly utilized in a credit scoring system.

### SMART

This section provides a detailed explanation of the concepts and methodologies employed for the imputation of missing values within a dataset.

To address the issue of missing values in incomplete dataset features, various imputation techniques can be employed. These include statistical methods like mean imputation (a form of simple imputation), machine learning methods such as Multiple Imputation by Chained Equations (MICE), K-Nearest Neighbors (KNN), MissForest, and neural network approaches like Generative Adversarial Imputation Networks (GAIN). The effectiveness of these imputation strategies will be evaluated in comparison to the proposed model, SMART, to assess whether it demonstrates robustness relative to established benchmarks, including GAIN-based methods, MissForest, MICE, and simple imputation techniques.

Prior to the application of randomized Singular Value Decomposition (rSVD) for noise reduction in the dataset, it is advisable to normalize the dataset. This normalization process ensures that the training of Generative Adversarial Imputation Networks (GAIN) is less affected by the scale of features. Normalization rescales the values within the dataset to the interval [0,1], thereby improving the performance of data synthesis by GAIN. Given that GAIN, being a neural network-based algorithm, is sensitive to the scale of input values during weight adjustment, normalization plays a crucial role. The normalization process is defined as follows:

5 $$\begin{aligned} Normalised(V_{i}) = \frac{V_{i} - min(V_{i})}{max(V_{i}) - min(V_{i})}, \end{aligned}$$

where $V_{i}$ represents the value of a feature, and $max(V_{i})$ and $min(V_{i})$ denote the maximum and minimum values of that feature, respectively.

After normalizing the dataset values, randomized Singular Value Decomposition (rSVD) is applied to further denoise the dataset. This step enhances the performance of the Generative Adversarial Imputation Networks (GAIN) in imputing missing values by effectively isolating key data structures and reducing redundancies, thus allowing GAIN to better model the underlying data distribution. The presence of noise and redundant features in real-world credit scoring datasets is common^42^.

**Figure 2:**
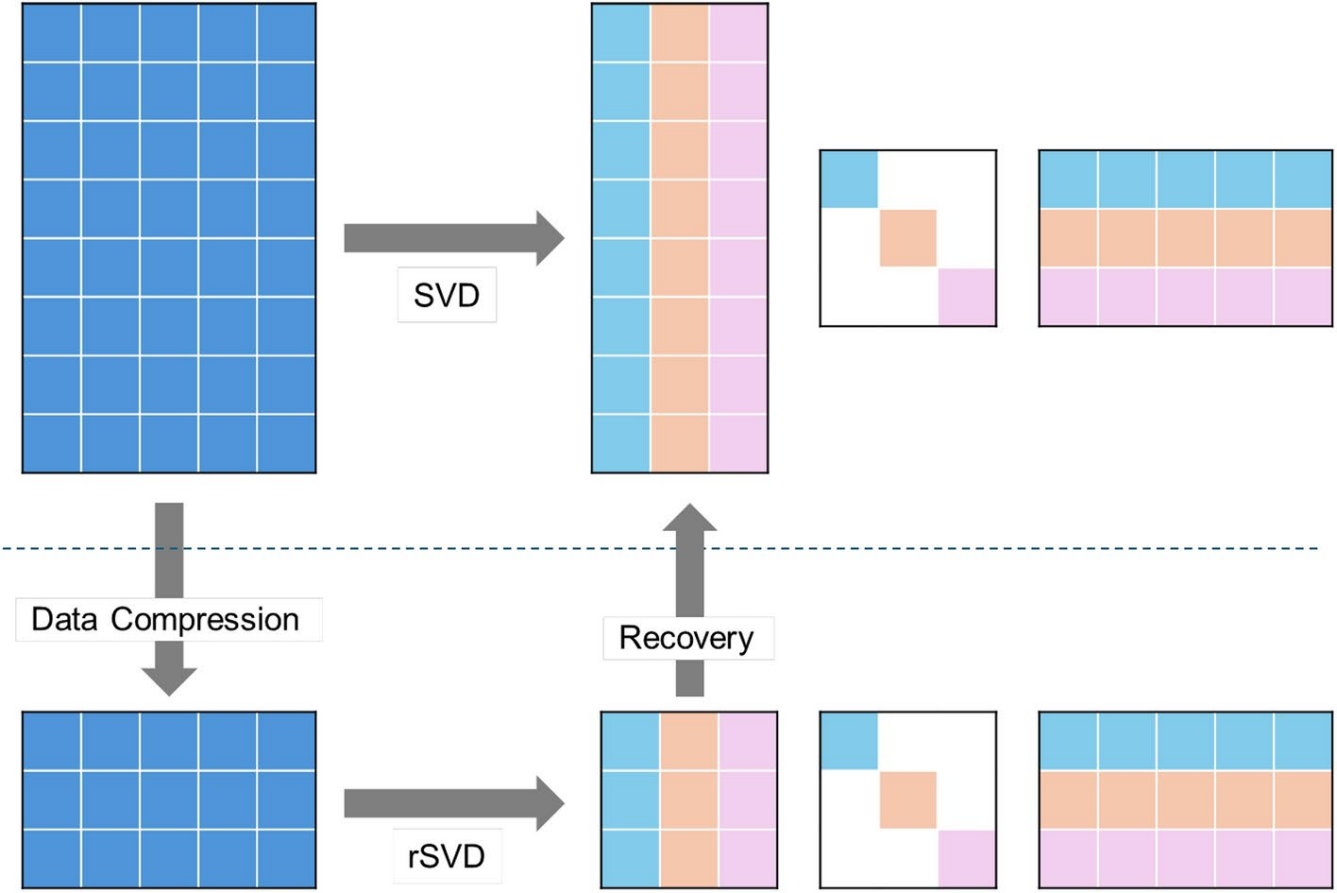
The conceptual architecture of rSVD.

Singular Value Decomposition (SVD) is a prevalent technique for matrix decomposition, frequently utilized for dimensionality reduction, noise suppression, and data analysis or compression, especially within the realm of image processing. The application of SVD enables the generation of a compressed image that exhibits reduced noise and improved feature extraction.

SVD can also be employed for imputing missing values in tabular datasets, based on the premise that the dataset consists of noisy samples resulting from linear combinations of principal factors^13^. By performing SVD, principal factors, known as eigenvectors, can be extracted. These eigenvectors improve the GAIN algorithm by assisting in the detection of patterns and characteristics within the dataset, thereby enhancing the estimation of missing values.

Given a matrix $M \in \mathbb {R}^{m \times n}$ with $m \times n$, SVD can be expressed as follows:

6 $$\begin{aligned} M = U \Sigma V^{T}, \end{aligned}$$

where matrix $U = [u_{1}, \ldots , u_{m}] \in \mathbb {R}^{m \times m}$, matrix $V = [v_{1}, \ldots , v_{m}] \in \mathbb {R}^{n \times n}$, and *U* and *V* are orthogonal. The left singular vectors in *U* shows a basis for column space (the range) and the right singular vectors in *V* shows a basis for domain space (the row) of the matrix *M*^43^. The rectangular matrix $\Sigma \in \mathbb {R}^{m \times n}$ has the corresponding singular values $\sigma _{1}$ $\ge$ $\cdots$ $\ge$ $\sigma _{n}$ $\ge$ 0 in diagonal, representing the spectrum of data^43^. That is,

7 $$\begin{aligned} M = U \Sigma V^{T} = [u_{1}, \ldots , u_{m}] diag(\sigma _{1}, \ldots , \sigma _{n}) [v_{1}, \ldots , v_{m}]^{T} \end{aligned}$$

Figure 2 schematically illustrates the conceptual framework of the randomized Singular Value Decomposition (rSVD) process, highlighting both the data compression and recovery stages involved in low-rank matrix approximation. Unlike traditional SVD, which is often computationally demanding, rSVD introduces randomness to provide a faster approximation, making it especially suitable for large-scale datasets with high levels of noise and redundancy, as commonly seen in credit scoring data. By decomposing the matrix into principal components, rSVD effectively highlights underlying patterns while reducing the impact of noisy features, thereby enhancing the subsequent imputation process^44^. Moreover, research by^34^ and^22^ has shown that Generative Adversarial Imputation Networks (GAIN) achieve enhanced performance when implemented on normalized datasets. Consequently, using rSVD as a preprocessing step for noise reduction, followed by GAIN for imputation, is anticipated to yield superior imputation accuracy by leveraging the denoising capacity of rSVD along with the distribution-capturing capabilities of GAIN.

Subsequent to the rescaling and denoising processes facilitated by normalization and rSVD, SMART employs GAIN-based imputation to replace the missing values with generated data within the incomplete dataset.

Generative Adversarial Imputation Networks (GAIN)^1^ were proposed to address the problem of missing values in datasets by leveraging the architecture of Generative Adversarial Networks (GANs)^45^. Missing values are imputed based on the conditional distribution or joint distribution of the complete data matrix *X*, as previously discussed. Unlike methods such as Multiple Imputation by Chained Equations (MICE)^46^, which rely on the expectation of multiple imputations, GANs generate synthetic data through adversarial learning involving a generator and a discriminator, allowing for imputation using the distribution of synthetic data.

Specifically, discriminative models like MICE and MissForest learn a function that maps inputs *x* to outputs *y*, enabling them to fill missing values using the conditional probability *P*(*y*|*x*). In contrast, generative models such as Expectation-Maximization (EM), Denoising AutoEncoder (DAE), and GAIN learn a probability distribution of the data, allowing them to fill missing values using the joint probability *P*(*x*, *y*).

**Figure 3:**
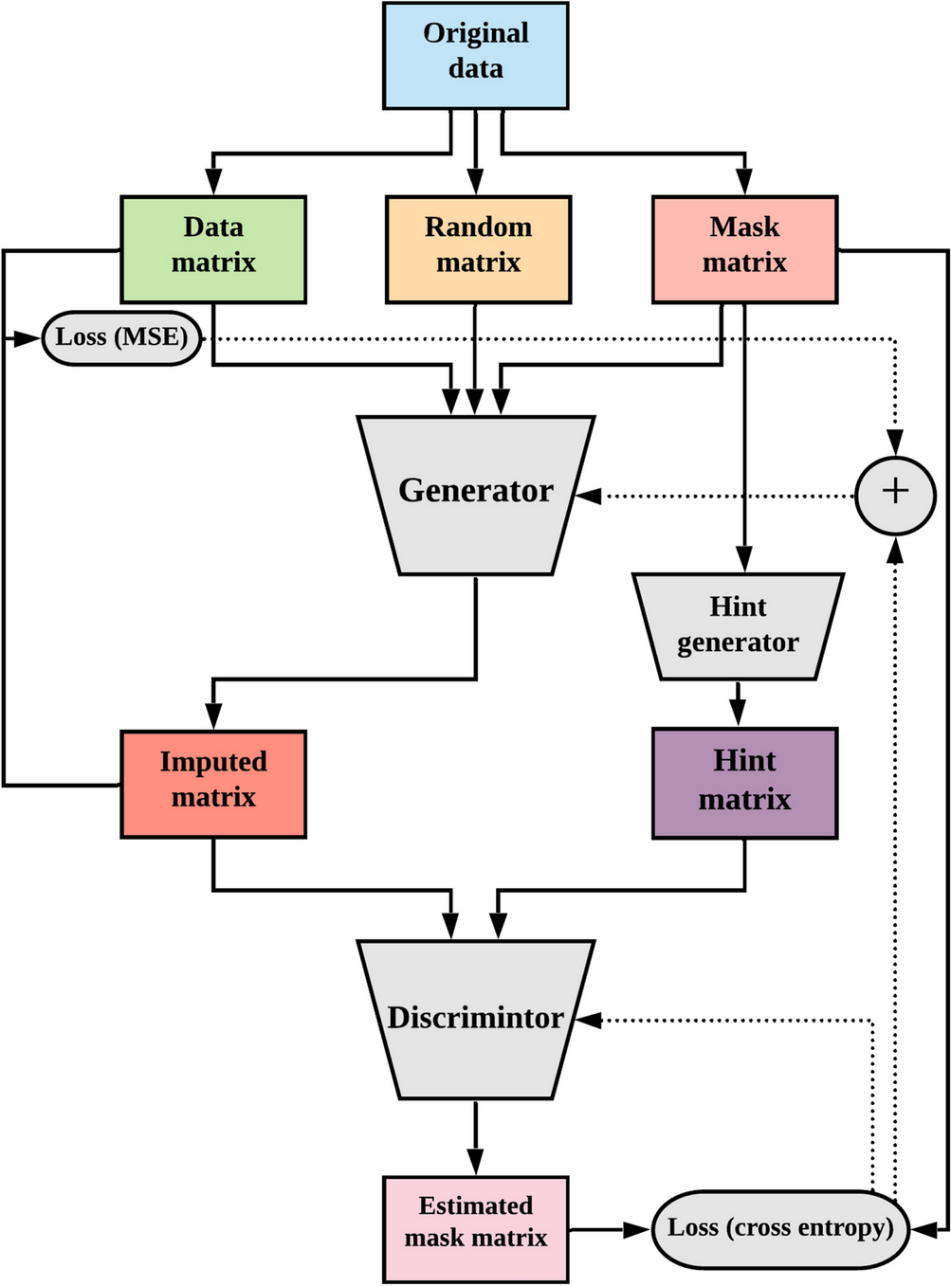
The architecture of GAIN.

To this end, the SMART methodology leverages the synergistic combination of randomized Singular Value Decomposition (rSVD) and Generative Adversarial Imputation Networks (GAIN) to enhance imputation accuracy, particularly in the presence of high missingness. The rSVD stage serves as a denoising mechanism that projects the high-dimensional, partially observed data onto a lower-dimensional subspace by capturing its dominant latent structure. This transformation reduces noise and preserves the underlying relationships among features, effectively recovering a cleaner manifold upon which the data approximately reside. Operating on this denoised and more structured representation enables the subsequent GAIN model to learn a more faithful approximation of the data’s conditional distribution. In contrast to traditional imputation methods such as MICE and MissForest, which lack explicit mechanisms for noise reduction or structural regularization, SMART exploits the low-rank characteristics of tabular credit data to guide imputation toward more coherent and data-consistent estimates.

Empirical and theoretical studies^47^ have shown that applying dimensionality reduction prior to imputation can enhance the performance of downstream models by emphasizing structurally meaningful patterns. Within SMART, the denoised representation obtained from rSVD provides an optimal input for the subsequent GAIN-based imputation, enabling the generative model to learn a more faithful approximation of the data distribution. This synergy effectively combines the advantages of linear structure extraction and non-linear generative modeling, resulting in more accurate and consistent imputations. The approach underscores the importance of preprocessing in generative imputation and provides a theoretically grounded justification for SMART’s architecture. This hybrid framework thus facilitates more accurate recovery of missing values, resulting in significantly improved imputation performance across a wide range of missingness levels.

The GAIN algorithm is derived from the architecture of GANs, incorporating both a generator *G* and a discriminator *D*, similar to standard GANs. However, GAIN exhibits distinct characteristics and objectives compared to traditional GANs. While the primary goal of a GAN is to generate a synthetic distribution from the original distribution and to identify whether the entire generated dataset is real or fake, the GAIN algorithm is designed to impute missing data in samples and to determine whether the generated values are real or fake. Specifically, the generator *G* in GAIN creates plausible components for the missing values in samples based on the distribution of the original data, whereas the discriminator *D* distinguishes between each generated (or imputed) component and the observed components in the data^1^.

In the GAIN algorithm, the generator *G* takes as inputs a data matrix, a random matrix, and a mask matrix. The mask matrix indicates whether a value is present or missing; a value present in the matrix is denoted by 1, while a missing value is denoted by 0. The discriminator *D* in GAIN predicts the complete estimated mask matrix, where each component indicates whether the corresponding input value is observed or missing in the original data. This prediction is facilitated by the hint matrix provided by the hint generator^25^. Figure 3 presents a schematic overview of the GAIN (Generative Adversarial Imputation Networks) architecture, detailing the interaction between the generator, discriminator, and auxiliary components such as the hint mechanism and mask matrices. This architecture highlights the adversarial training process used for imputing missing data, where both Mean Squared Error (MSE) and cross-entropy losses are employed to guide the generator and discriminator respectively.

To compare the GAN architecture with the GAIN architecture, the two-player minimax game in GAN can be represented by the following objective function:

8 $$\begin{aligned} \min _{G} \max _{D} V(D, G) = \mathbb {E}_{x \sim P_{data}(x)}[\log D(x)] + \mathbb {E}_{z \sim P_{z}(z)}[\log (1 - D(G(z)))] \end{aligned}$$

Based on the architecture of GAN, the objective function of GAIN can be expressed as follows:

9 $$\begin{aligned} \min _{G} \max _{D} V(D, G) = \mathbb {E}_{\hat{X}, M, H} \left[ M^{T} \log D(\hat{X}, H) + (1-M)^{T} \log (1 - D(\hat{X}, H)) \right] , \end{aligned}$$

where *M* is a mask matrix, which indicates the position of missing values in the original incomplete matrix *X*, and *H* is a hint matrix, which prevents the discriminator *D* from learning dominantly when compared to generator *G*, and $D(\hat{X}, H)=\hat{M}$ is the probability such that the imputed values are observed values.

Generator *G* takes inputs as data matrix $\tilde{X}$, random matrix *Z* and mask matrix *M*, and generates output as imputed matrix $\hat{X}$. Discriminator *D* takes inputs as imputed matrix $\hat{X}$ and hint matrix *H*, and generates output as an estimated mask matrix $\hat{M}$ = $D(\hat{X}, H)$. The objective of the generator *G* in GAIN is to produce plausible synthetic values that closely approximate the observed values where the mask matrix *M* indicates a 1 (missing), thereby deceiving the discriminator *D* into classifying the synthetic values as real or observed. Conversely, the objective of the discriminator *D* in GAIN is to accurately identify observed values where the mask matrix *M* indicates a 1 (observed) and to correctly distinguish missing values where the mask matrix *M* indicates a 0 (missing).

**Figure 4:**
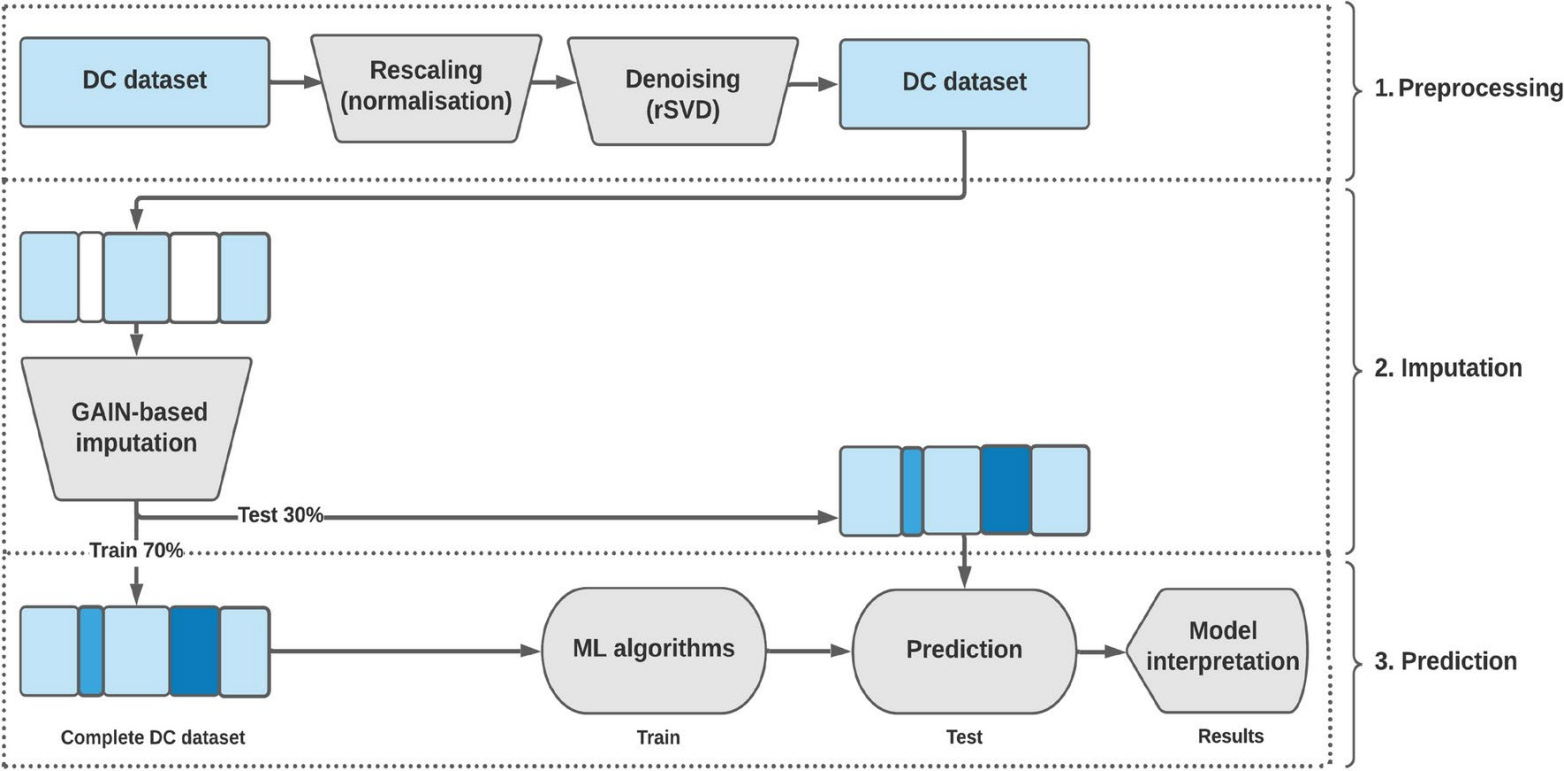
The system architecture of SMART.

This process in GAIN also constitutes a two-player minimax game, as discussed earlier.

The loss of the generator *G* in GAIN consists of two components since *G* outputs the imputed matrix for both observed and missing values. The first part represents the loss associated with the imputed (missing) values, while the second part represents the loss associated with the observed values^25^. Therefore, the loss function of the generator *G* in GAIN can be expressed as follows:

10 $$\begin{aligned} L_{G}(m, \hat{m}, b) = -\sum _{i : b_{i}} (1 - m_{i}) \log (\hat{m}_{i}) + \alpha \sum _{j=1}^{d} m_{j} L_{M}(x_{j}, \hat{x}_{j}) \end{aligned}$$

The first term captures the loss for the imputed (missing) values, encouraging the generator to produce plausible values, while the second term captures the loss for the observed values, ensuring consistency with the actual observed data. Here, $\alpha$ denotes a positive hyperparameter and $L_{M}(x_{i}, \hat{x_{i}})$ is as follows^1^:

11 $$\begin{aligned} L_{M}(x_{i}, \hat{x_{i}}) = {\left\{ \begin{array}{ll} m_{i}(\hat{x_i} - x_{i})^{2}, & \text {if}\ x_{i}\ \text {is continuous} \\ m_{i}(-x_{i}log(\hat{x_{i}})), & \text {if}\ x_{i} \ \text {is binary} \end{array}\right. } \end{aligned}$$

The loss of the discriminator *D* in GAIN can also be expressed in the form of cross-entropy as follows:

12 $$\begin{aligned} L_{D}(m, \hat{m}, b) = \sum _{i:b_{i}}(m_{i}log(\hat{m_{i}}) + (1-m_{i})log(1-\hat{m_{i}})) \end{aligned}$$

This loss function combines the cross-entropy losses for both observed and imputed (missing) values, guiding the discriminator to correctly identify observed values and distinguish them from imputed values. Here, $b_{i}$ is the $i^{th}$ element of a random variable *B* = $(B_{1},..., B_{d})\in \{0, 1\}^{d}$, which is acquired by sampling *k* from {1, ..., *d*} uniformly at random and $m_{i}$ is the $i^{th}$ element of mask matrix *M*^1^. And $B_{j}$ is as follows:

13 $$\begin{aligned} B_{j}=\left\{ \begin{array}{ll} 1, & \text {if } j \ne k \\ 0, & \text {if } j = k \\ \end{array}\right. \end{aligned}$$

The imputation performance of SMART is evaluated in comparison to several benchmarks, including MissForest, a machine learning-based random forest imputation method, and MICE, a machine learning-based regression tree imputation method. Furthermore, the assessment includes various variants of GAIN approaches.

Following the imputation of values generated by SMART, the model can subsequently utilize the complete dataset to conduct credit scoring predictions. Logistic Regression (LR) is a widely adopted technique for assessing the performance of imputation methods for missing data^2,3^ and is regarded as a versatile model for credit scoring^17,48,49^. Figure 4 illustrates the overall system architecture of the SMART framework, delineating its three main stages: preprocessing, imputation, and prediction. This diagram provides a high-level schematic of the data flow, beginning with normalization and denoising procedures, followed by GAIN-based imputation, and concluding with the training, evaluation, and interpretation of predictive models. The structure is intended to clarify the sequential dependencies and modular design of the framework.

**Table 2:** RMSE comparison for imputation performance of the proposed SMART against the benchmarks on default credit card with 5%, 10%, 15%, 20%, 50%, 80% missing data (lowest RMSE highlighted in bold).

Method	5%	10%	15%	20%	50%	80%
SMART	**0.0724**±**0.0009**	**0.0763**±**0.0007**	**0.0818**±**0.0033**	**0.0857**±**0.0005**	**0.1292**±**0.0068**	**0.1449**±**0.0199**
GAIN+vs^*^^34^	N/A	N/A	N/A	0.1190±0.004	0.1840±0.004	0.2550±0.003
GAIN^34^	N/A	N/A	N/A	0.1230±0.001	0.1940±0.002	0.2700±0.005
SGAIN^36^	N/A	N/A	N/A	0.157	N/A	N/A
WSGAIN-CP^36^	N/A	N/A	N/A	0.163	N/A	N/A
WSGAIN-GP^36^	N/A	N/A	N/A	0.165	N/A	N/A
CGAIN^25^	0.2329±0.0039	0.2009±0.0022	0.2314±0.0035	0.2213±0.0099	N/A	N/A
MissForest^25^	0.2902±0.0010	0.2439±0.0079	0.2672±0.0025	0.2646±0.0026	N/A	N/A
MICE^25^	0.2479±0.0079	0.2491±0.0085	0.2479±0.0074	0.2480±0.0091	N/A	N/A

^*^ Variables split

**Figure 5:**
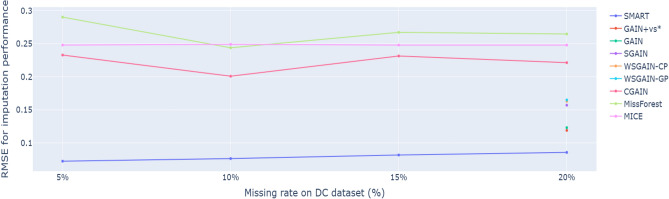
RMSE comparison for imputation performance of the proposed SMART against the GAIN-based and machine learning imputation methods on default credit card with 5%, 10%, 15% and 20% missing data.

**Figure 6:**
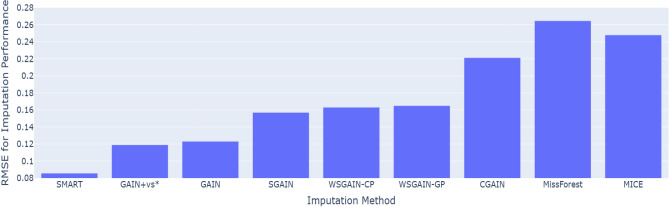
RMSE comparison for imputation performance of the proposed SMART against the GAIN-based and machine learning imputation methods on default credit card with 20% missing data.

## Results

This section assesses the imputation and prediction performance of SMART. To validate the efficacy of SMART for addressing missing values or incomplete data, missing values are randomly introduced at rates of 5%, 10%, 15%, 20%, 50%, and 80%. The removal of values from the dataset operates under the assumption of Missing Completely at Random (MCAR) within the complete dataset.

### Experimental setup

The imputation performance of SMART is assessed using the Root Mean Square Error (RMSE) metric to validate its imputation accuracy. RMSE is calculated as the square root of the Mean Squared Error (MSE) for the prediction *f*(*x*) relative to the true label *y*. The MSE is defined as follows:

14 $$\begin{aligned} \text {MSE}(f(x)) = \mathbb {E}[(f(x) - y)^{2}] = \frac{1}{m} \sum _{i=1}^{m} (f(x_{i}) - y_{i})^{2} = \int _{x \sim D} (f(x) - y)^{2} p(x) \, dx, \end{aligned}$$

where *D* is the distribution of data and $p(\cdot )$ is the probability density function.

Prior to assessing the classification performance resulting from imputation, it is imperative to first evaluate the imputation effectiveness of the SMART framework on the DC dataset. The proposed SMART method is benchmarked against state-of-the-art imputation methods, including several variants of GAIN —GAIN+vs^34^, GAIN^34^, SGAIN^36^, WSGAIN-CP^36^, WSGAIN-GP^36^, CGAIN^25^— as well as standard machine learning-based imputation methods such as MissForest^25^ and MICE^25^.

To identify the optimal hyperparameter settings for the GAIN model, we performed an extensive grid search in conjunction with 5-fold cross-validation. This approach was aimed at ensuring stable and generalizable imputation performance across a range of parameter configurations. Specifically, we tuned the following hyperparameters: the reconstruction loss weight ($\alpha$), learning rate, number of training epochs, mini-batch size, missing data rate, hint rate, and the proportion of training data. Each hyperparameter combination was systematically assessed through 5-fold cross-validation, wherein the RMSE and its standard deviation were computed across the folds. The configuration achieving the lowest average RMSE was adopted as the optimal setting to ensure a robust and principled determination of model parameters.

Following the identification of the optimal hyperparameters, we further evaluated the predictive capability of the GAIN model through an additional 5-fold cross-validation, using the Area Under the Receiver Operating Characteristic Curve (AUROC) as the evaluation metric. AUROC is widely recognized as a robust and informative criterion for assessing classification performance, particularly in the presence of class imbalance^48–51^. Unlike overall classification accuracy, which may be biased toward the majority class, AUROC provides a balanced and comprehensive evaluation of a model’s predictive performance across class distributions. As such, it is regarded as a standard and reliable indicator in imbalanced classification tasks^48,49^.

To further ensure the reliability and reproducibility of our results, we applied 5-fold cross-validation consistently across all imputation and classification experiments. In each fold, the dataset was partitioned into distinct training and test sets while preserving consistent patterns of missingness. Moreover, the entire experimental procedure was repeated over two independent runs with different random seeds, thereby accounting for variations due to random initialization and sample selection. This comprehensive evaluation enabled us to assess both the stability and generalizability of the proposed approach under diverse experimental settings.

**Figure 7:**
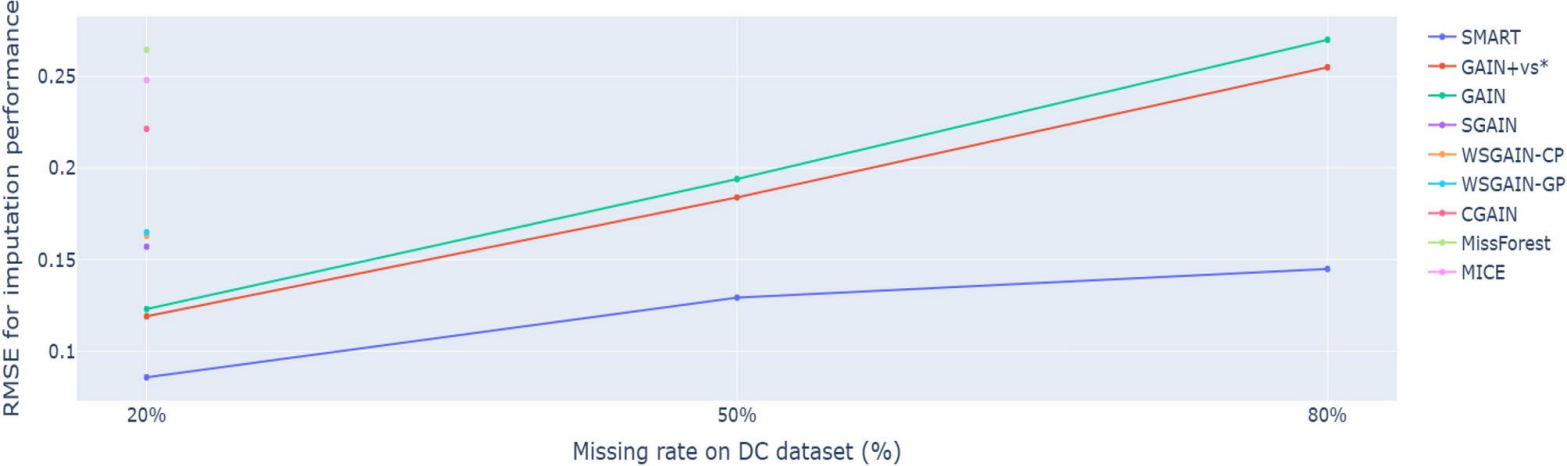
RMSE comparison for imputation performance of the proposed SMART against the GAIN-based and machine learning imputation methods on default credit card with 20%, 50% and 80% missing data.

### The imputation performance

Table 2 summarizes the RMSE-based imputation performance of the proposed SMART framework and benchmark methods across different levels of missingness (5%, 10%, 15%, 20%, 50%, and 80%) in the default credit card dataset. Complementarily, Fig. 5 illustrates the comparative performance trends under lower missingness scenarios (5%, 10%, 15%, and 20%), providing a visual representation of the models’ robustness in recovering missing values. Across all evaluated levels of missing data, the proposed SMART framework consistently yielded the lowest RMSE, demonstrating superior imputation accuracy relative to all benchmark methods. In particular, SMART outperformed both GAN-based methods-such as GAIN+vs^34^, GAIN^34^, SGAIN^36^, WSGAIN-CP^36^, WSGAIN-GP^36^, CGAIN^25^ as well as traditional machine learning approaches including MissForest^25^ and MICE^25^. These results highlight the effectiveness of SMART’s hybrid design, which combines rSVD-based denoising with GAIN-based imputation to better preserve underlying data structure and mitigate the impact of missingness. The consistently lower RMSE scores affirm SMART’s robustness and precision in recovering missing data in credit scoring tasks under moderate missingness conditions.

Figure 6 presents a comparative analysis of imputation performance, measured by RMSE, for the proposed SMART framework against various GAIN-based and traditional machine learning imputation methods on the default credit card dataset with 20% missingness. At this level, the proposed SMART framework demonstrated superior performance, achieving the lowest RMSE (0.0857±0.0005) among all evaluated methods. Notably, SMART outperformed several benchmark imputation techniques, including GAIN+vs^34^, GAIN^34^ SGAIN^36^, WSGAIN-CP^36^, WSGAIN-GP^36^, CGAIN^25^, MissForest^25^, and MICE^25^. These results underscore the effectiveness of SMART’s hybrid approach, which integrates rSVD-based denoising with GAIN-based imputation to preserve structural data integrity. Furthermore, the consistent performance advantage over both GAN-based and traditional machine learning-based imputers highlights SMART’s robustness in handling the level of missingness in credit scoring dataset.

Figure 7 presents a comparative assessment of the SMART framework against GAIN-based and traditional imputation methods in terms of RMSE, evaluated on the default credit card dataset with 20%, 50%, and 80% missingness levels. The proposed SMART method consistently demonstrated the highest imputation performance across these scenarios. Furthermore, SMART exhibited superior performance under extreme conditions, effectively handling datasets with 80% missing values and outperforming all benchmark models.

As expected, the imputation performance of all GAIN-based models declines with an increase in the percentage of missing data. Nevertheless, GAIN-based imputation approaches consistently outperformed machine learning-based imputation methods, as demonstrated in Fig. 5. This suggests that GAIN-based models possess robust imputation capabilities, as they are continuously updated through feedback using the cross-entropy loss from the discriminator and the observed values in the dataset. Consequently, GAIN-based models demonstrate the ability to withstand high proportions of missing data. For this reason, SMART exhibited stable performance even as the proportion of missing data increased, as shown in Fig. 7.

**Table 3:** AUROC comparison for Prediction performance of the proposed SMART against the benchmarks of GAIN-based prediction on default credit card with 20% missing data (best AUROC highlighted in bold).

Method	20%
SMART	**0.7215**
SGAIN^36^	0.7112
WSGAIN-GP^36^	0.7096
GAIN^36^	0.7095
WSGAIN-CP^36^	0.7094

**Figure 8:**
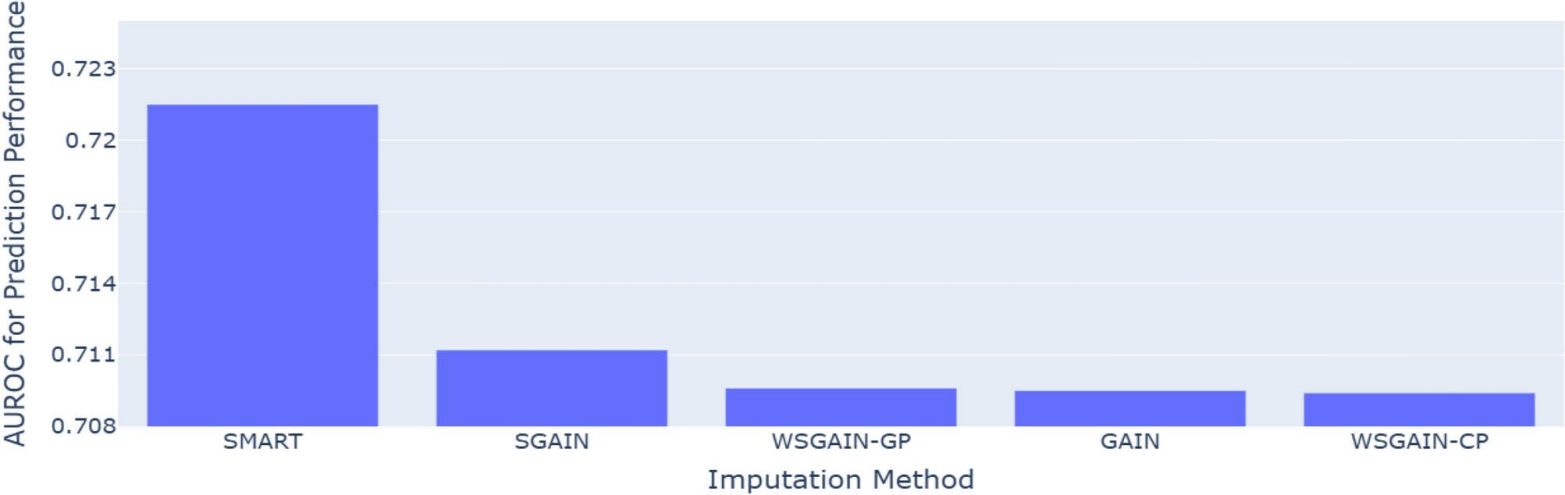
AUROC comparison for prediction performance of the proposed SMART against the GAIN-based imputation methods on default credit card with 20% missing data.

Therefore, the enhancement of GAIN imputation combined with rSVD was demonstrated by improving imputation performance on an incomplete credit scoring dataset. This improvement was evident when compared to benchmarks, including the original GAIN, its variants, and conventional statistical and machine learning imputation methods. The superior imputation capability of SMART is expected to play a pivotal role in enhancing the predictive performance of credit scoring models. This capability allows downstream classifiers to operate on more complete and structurally coherent data. Specifically, by integrating rSVD-based denoising with GAIN-based imputation, SMART effectively reconstructs missing values while preserving the latent relationships among features that are critical for accurate default risk modeling. This improvement in data quality significantly enhances the reliability of credit risk estimation. As demonstrated by post-imputation classification outcomes in prior studies^1,12,41,52^, imputations that are both structure-aware and noise-reduced contribute meaningfully to the overall predictive performance of credit scoring systems. These findings underscore the importance of high-fidelity imputation in supporting robust and generalizable risk prediction models. Ultimately, SMART successfully introduced a novel architecture for credit scoring models designed to handle datasets with missing values.

### The prediction performance

Given the class imbalance inherent in the DC dataset, the Area Under the Receiver Operating Characteristic Curve (AUROC) is employed as the evaluation metric, as it is widely recognized as the standard for assessing classification performance in imbalanced settings^48–51^. As discussed earlier, Logistic Regression (LR) is adopted for evaluating predictive performance, owing to its versatility and widespread use in credit scoring applications^17,48,49^. In this study, LR is used to distinguish between good and bad credit applicants, and the predictive performance of SMART is compared against several state-of-the-art imputation methods under a 20% missing data condition.

Table 3 and Fig. 8 presents a comparative evaluation of prediction performance, measured by AUROC, between the proposed SMART framework and various GAIN-based imputation methods. The proposed SMART framework outperforms benchmark methods, demonstrating the highest classification performance. This performance gain is attributed to SMART’s superior imputation strategy, which integrates rSVD-based denoising with GAIN-based reconstruction to preserve latent feature relationships critical for default risk modeling. By improving the quality of input data, SMART facilitates more reliable credit risk estimation and highlights the critical role of imputation in constructing robust and generalizable predictive models for practical financial applications.

**Table 4:** Friedman test results for imputation performance (p < 0.05 highlighted in bold).

Table 2	Statistic (Chi-Square)	p-value	Significance
Imputation performance (RMSE)	34.81	**0.0001**	Significant

### Statistical evaluation of the imputation performance in the SMART framework

The Friedman test was employed to evaluate the performance of the SMART framework, owing to its suitability for analyzing repeated measures across varying levels of missingness. As a non-parametric statistical method, the Friedman test is particularly appropriate in scenarios involving multiple treatments applied to the same datasets^53^. In this study, the test was used to examine whether observed differences in imputation performance-measured by RMSE-among competing methods were statistically significant. By ranking the RMSE scores across imputation techniques and across different missingness levels, the Friedman test enabled a rigorous comparison. This analysis provided a statistically sound foundation for validating that the improvements achieved by the SMART framework are meaningful when compared to alternative imputation approaches.

The results presented in Table 4 summarize the outcomes of the Friedman test, which was conducted to assess whether the differences in imputation performance across competing methods are statistically significant. The comparison includes SMART and a suite of benchmark techniques, such as GAIN+vs, GAIN, SGAIN, WSGAIN-CP, WSGAIN-GP, CGAIN, MissForest, and MICE. To facilitate the Friedman test on the data summarized in Table 2, missing values originally reported as “N/A” in the references were imputed under a conservative assumption: that each method exhibits a monotonic increase in RMSE as the proportion of missing data increases. This assumption allows for consistent inclusion of all methods in the statistical evaluation.

The resulting chi-square statistic of 34.81 and the associated p-value of 0.0001 (p< 0.05) confirm that the differences in RMSE among the evaluated imputation methods are statistically significant. These findings provide strong empirical support for the claim that SMART delivers superior imputation performance. Notably, SMART consistently achieved the lowest RMSE across a wide range of missingness levels, outperforming both conventional machine learning techniques and advanced generative models. Overall, the Friedman test results reinforce the effectiveness of SMART in producing structurally coherent and statistically reliable imputations. These findings underscore SMART’s practical utility for handling missing data in real-world credit scoring applications, where data quality is critical to predictive performance.

## Discussion

Generative Adversarial Imputation Networks (GAIN), grounded in the architecture of GANs, have shown considerable effectiveness in generating synthetic data, particularly for imputing missing values. GAIN’s ability to learn and replicate complex data distributions makes it a promising approach for handling missing values in synthetic tabular datasets. However, despite this potential, GAIN faces several challenges when applied to tabular data, which typically comprises features with widely differing distribution patterns, intricate interdependencies, and varying degrees of sparsity.

One prominent challenge in training GANs is mode collapse, a phenomenon wherein the generator fails to capture the full diversity of the data distribution^36,54–56^. In the context of tabular data, mode collapse can result in synthetic outputs that do not adequately represent the range of values in the original dataset^48^. For instance, in a credit scoring dataset, mode collapse may lead to synthetic values that reflect only a subset of the overall distribution, thus limiting the usefulness of the imputed data. Mode collapse is often identified by the generation of overly similar outputs or a flattened generator loss curve, indicating incomplete learning of the data distribution^48^. Several techniques have been proposed to address mode collapse, such as minibatch discrimination and spectral normalization^54,55^. Minibatch discrimination encourages diversity in the generator’s outputs by enabling the discriminator to assess patterns across batches of data, rather than isolated samples. Spectral normalization, meanwhile, stabilizes GAN training by controlling the Lipschitz constant of the discriminator, helping the generator to learn meaningful features and mitigate mode collapse. Together, these techniques enhance the generator’s ability to produce outputs that more accurately represent the original data distribution^54,55^.

In addition to mode collapse, training instability presents another significant challenge in employing GANs for tabular data generation^36,48,54–56^. Training instability arises from the adversarial dynamics between the generator and discriminator, with each network continually adapting to outperform the other. This competitive interaction can result in oscillating loss curves without convergence, ultimately producing low-quality synthetic data^48^. In the context of imputation, such instability may generate unrealistic or nonsensical values for missing data that fail to align with the logical or statistical properties of the original dataset. Training instability can be further aggravated by factors such as vanishing gradients, which limit feedback to the generator from the discriminator, or by inadequate initialization and suboptimal hyperparameter settings, leading to erratic training behavior^48,54,55^.

Given that credit scoring datasets often encompass diverse data types, including both numerical and categorical features, and recognizing that numerical values may exhibit complex distributions, such as multi-modal or heavy-tailed patterns, generating both numerical and categorical data simultaneously using GANs presents a unique challenge^48,52^. Strategies aimed at addressing the instability in GAN training, such as modifying the loss function or introducing novel stability-enhancing techniques, have the potential to facilitate more reliable and realistic data generation, particularly in the context of imputing missing values.

While the results obtained from the Default of Credit Card Clients (DC) dataset are promising, we acknowledge that limiting the evaluation of the SMART framework to a single dataset constitutes a limitation of the present study. To address this, we have emphasized the necessity of validating SMART across a broader range of credit scoring datasets to enhance the generalizability of our findings. In addition to exploring the integration of generative models for imputing missing data, we recognize the importance of systematically assessing the framework’s applicability in diverse contexts. Accordingly, future research will extend the evaluation to multiple publicly available credit scoring datasets, including the German, Australian, Taiwanese, and Polish datasets from the UCI Machine Learning Repository. This expanded empirical analysis will facilitate a more comprehensive understanding of SMART’s robustness, scalability, and practical relevance across varying data characteristics and credit scoring environments.

## Conclusion

This study introduced SMART, a novel imputation framework designed to address the pervasive issue of missing values in credit scoring datasets. The methodology builds upon and extends the state-of-the-art Generative Adversarial Imputation Network (GAIN), with particular emphasis on enhancing the generative modeling process through the integration of data normalization and randomized Singular Value Decomposition (rSVD). Empirical evaluations demonstrated that SMART effectively reconstructs missing values by leveraging the latent structure of the dataset, thereby outperforming traditional machine learning approaches such as MICE and MissForest, as well as the original GAIN model and its advanced variants, including CGAIN, SGAIN, WSGAIN-CP, and WSGAIN-GP. Notably, the improved imputation quality achieved by SMART translates into superior predictive performance in downstream credit scoring tasks. These results highlight SMART’s suitability for applications involving complex tabular data where accurate estimation of missing values is critical.

Future research could aim to validate the generalizability of SMART across a broader range of credit scoring datasets and regulatory contexts, which is essential for ensuring its readiness for real-world deployment. Furthermore, the core principles of the SMART framework hold promise for adaptation to other complex data domains, such as natural language processing, speech recognition, and image-based imputation tasks, thereby expanding its applicability beyond the financial sector.

## Data Availability

The ‘Default of Credit Card Clients (DC)’ dataset is accessible via UCI Machine Learning Repository at the following: https://archive.ics.uci.edu/dataset/350/default+of+credit+card+clients.

## References

[1] Yoon, J., Jordon, J. & Schaar, M. Gain: Missing data imputation using generative adversarial nets. In *International Conference on Machine Learning*, 5689–5698 (PMLR, 2018).

[2] Horton, N. J. & Kleinman, K. P. Much ado about nothing: A comparison of missing data methods and software to fit incomplete data regression models. *Am. Stat.* **61**, 79–90 (2007). 17401454 10.1198/000313007X172556 PMC1839993

[3] Ibrahim, J. G., Chen, M.-H., Lipsitz, S. R. & Herring, A. H. Missing-data methods for generalized linear models: A comparative review. *J. Am. Stat. Assoc.* **100**, 332–346 (2005).

[4] García-Laencina, P. J., Sancho-Gómez, J.-L. & Figueiras-Vidal, A. R. Pattern classification with missing data: a review. *Neural Comput. Appl.* **19**, 263–282 (2010).

[5] Li, P., Stuart, E. A. & Allison, D. B. Multiple imputation: a flexible tool for handling missing data. *JAMA* **314**, 1966–1967 (2015). 26547468 10.1001/jama.2015.15281 PMC4638176

[6] Little, R. & Rubin, D. B. *Statistical Analysis with Missing Data* (John Wiley & Sons, Hoboken, NJ, 2019) (**3rd edn**).

[7] Schafer, J. L. *Analysis of Incomplete Multivariate Data* (CRC Press, 1997) (**1st edn.**).

[8] McKnight, P. E., McKnight, K. M., Sidani, S. & Figueredo, A. J. *Missing Data: A Gentle Introduction* (Guilford Press, 2007) (**1st edn.**).

[9] Jerez, J. M. et al. Missing data imputation using statistical and machine learning methods in a real breast cancer problem. *Artif. Intell. Med.* **50**, 105–115 (2010). 20638252 10.1016/j.artmed.2010.05.002

[10] Zhao, F. et al. Multiple imputation method of missing credit risk assessment data based on generative adversarial networks. *Appl. Soft Comput.* **126**, 109273 (2022).

[11] Ruiz-Chavez, Z., Salvador-Meneses, J. & Garcia-Rodriguez, J. Machine learning methods based preprocessing to improve categorical data classification. In *International Conference on Intelligent Data Engineering and Automated Learning*, 297–304 (Springer, 2018).

[12] Śmieja, M., Struski, Ł., Tabor, J., Zieliński, B. & Spurek, P. Processing of missing data by neural networks. *Adv. Neural Inf. Process. Syst.* **31** (2018).

[13] Bertsimas, D., Pawlowski, C. & Zhuo, Y. D. From predictive methods to missing data imputation: an optimization approach. *J. Mach. Learn. Res.* **18**, 7133–7171 (2017).

[14] Nationalbank, O. *Guidelines on credit risk management: Rating models and validation* (Oesterreichische Nationalbank, 2004).

[15] Kline, R. B. *Principles and Practice of Structural Equation Modeling* (Guilford Press, New York, NY, 2015) (**4th edn.**).

[16] Salgado, C. M., Azevedo, C., Proença, H. & Vieira, S. M. Missing data. *Secondary analysis of electronic health records* 143–162 (2016).

[17] Florez-Lopez, R. Effects of missing data in credit risk scoring. a comparative analysis of methods to achieve robustness in the absence of sufficient data. *J. Oper. Res. Soc.* **61**, 486–501 (2010).

[18] Shahbazian, R. & Greco, S. Generative adversarial networks assist missing data imputation: a comprehensive survey and evaluation. *IEEE Access* **11**, 88908–88928 (2023).

[19] Buuren, S. v. & Groothuis-Oudshoorn, K. mice: Multivariate imputation by chained equations in r. *J. Stat. Softw.* 1–68 (2010).

[20] Stekhoven, D. J. & Bühlmann, P. Missforest–non-parametric missing value imputation for mixed-type data. *Bioinformatics* **28**, 112–118 (2012). 22039212 10.1093/bioinformatics/btr597

[21] Little, R. J. & Rubin, D. B. The analysis of social science data with missing values. *Sociol. Methods Res.* **18**, 292–326 (1989).

[22] Hammad Alharbi, H. & Kimura, M. Missing data imputation using data generated by gan. In *2020 the 3rd International Conference on Computing and Big Data*, 73–77 (2020).

[23] Vriens, M. & Melton, E. Managing missing data. *Mark. Res.* **14**, 12 (2002).

[24] Baraldi, A. N. & Enders, C. K. An introduction to modern missing data analyses. *J. Sch. Psychol.* **48**, 5–37 (2010). 20006986 10.1016/j.jsp.2009.10.001

[25] Awan, S. E., Bennamoun, M., Sohel, F., Sanfilippo, F. & Dwivedi, G. Imputation of missing data with class imbalance using conditional generative adversarial networks. *Neurocomputing* **453**, 164–171 (2021).

[26] Dempster, A. P., Laird, N. M. & Rubin, D. B. Maximum likelihood from incomplete data via the em algorithm. *J. Roy. Stat. Soc.: Ser. B (Methodol.)* **39**, 1–22 (1977).

[27] Yuan, Y. C. Multiple imputation for missing data: Concepts and new development (version 9.0). *SAS Institute Inc, Rockville, MD* **49**, 12 (2010).

[28] Dong, W. et al. Generative adversarial networks for imputing missing data for big data clinical research. *BMC Med. Res. Methodol.* **21**, 1–10 (2021). 33879090 10.1186/s12874-021-01272-3 PMC8059005

[29] Seaman, S. R., Bartlett, J. W. & White, I. R. Multiple imputation of missing covariates with non-linear effects and interactions: an evaluation of statistical methods. *BMC Med. Res. Methodol.* **12**, 1–13 (2012). 22489953 10.1186/1471-2288-12-46 PMC3403931

[30] Troyanskaya, O. et al. Missing value estimation methods for dna microarrays. *Bioinformatics* **17**, 520–525 (2001). 11395428 10.1093/bioinformatics/17.6.520

[31] Shah, A. D., Bartlett, J. W., Carpenter, J., Nicholas, O. & Hemingway, H. Comparison of random forest and parametric imputation models for imputing missing data using mice: a caliber study. *Am. J. Epidemiol.* **179**, 764–774 (2014). 24589914 10.1093/aje/kwt312 PMC3939843

[32] Honaker, J. et al. Amelia ii: A program for missing data. *J. Stat. Softw.* **45**, 1–47 (2011).

[33] Nazabal, A., Olmos, P. M., Ghahramani, Z. & Valera, I. Handling incomplete heterogeneous data using vaes. *Pattern Recogn.* **107**, 107501 (2020).

[34] Camino, R. D., Hammerschmidt, C. A. & State, R. Improving missing data imputation with deep generative models. ArXiv preprint, arxiv: 1902.10666v1 (2019).

[35] Friedjungová, M., Vašata, D., Balatsko, M. & Jiřina, M. Missing features reconstruction using a wasserstein generative adversarial imputation network. In *International Conference on Computational Science*, 225–239 (Springer, 2020).

[36] Neves, D. T., Naik, M. G. & Proença, A. Sgain, wsgain-cp and wsgain-gp: Novel gan methods for missing data imputation. In *International Conference on Computational Science*, 98–113 (Springer, 2021).

[37] Rubin, D. B. Inference and missing data. *Biometrika* **63**, 581–592 (1976).

[38] Schafer, J. L. & Graham, J. W. Missing data: our view of the state of the art. *Psychol. Methods* **7**, 147 (2002). 12090408

[39] Enders, C. K. & Gottschall, A. C. Multiple imputation strategies for multiple group structural equation models. *Struct. Equ. Model.* **18**, 35–54 (2011).

[40] Yeh, I.-C. & Lien, C.-H. The comparisons of data mining techniques for the predictive accuracy of probability of default of credit card clients. *Expert Syst. Appl.* **36**, 2473–2480 (2009).

[41] Halmich, C. *WGAIN: Data Imputation Using Wasserstein GAIN*. Master’s thesis, Johannes Kepler University (2020). Master’s Program in Bioinformatics.

[42] Xia, Y., Liu, C., Li, Y. & Liu, N. A boosted decision tree approach using bayesian hyper-parameter optimization for credit scoring. *Expert Syst. Appl.* **78**, 225–241 (2017).

[43] Erichson, N. B., Voronin, S., Brunton, S. L. & Kutz, J. N. Randomized matrix decompositions using r. *J. Stat. Softw.* **89**, 1–48 (2019).

[44] Erichson, N. B., Mathelin, L., Kutz, J. N. & Brunton, S. L. Randomized dynamic mode decomposition. *SIAM J. Appl. Dyn. Syst.* **18**, 1867–1891 (2019).

[45] Goodfellow, I. et al. Generative adversarial nets. *Adv. Neural. Inf. Process. Syst.* **27**, 2672–2680 (2014).

[46] Royston, P. et al. Multiple imputation by chained equations (mice): implementation in stata. *J. Stat. Softw.* **45**, 1–20 (2011).

[47] Nguyen, M. *A Guide on Data Analysis: From Basics to Causal Inference* (Bookdown, 2020) (**1st edn.**).

[48] Han, S., Jung, H., Yoo, P. D., Provetti, A. & Cali, A. Note: non-parametric oversampling technique for explainable credit scoring. *Sci. Rep.* **14**, 26070 (2024). 39478045 10.1038/s41598-024-78055-5 PMC11525592

[49] Han, S. & Jung, H. Nate: Non-parametric approach for explainable credit scoring on imbalanced class. *PLoS ONE* **19**, e0316454 (2024). 39739883 10.1371/journal.pone.0316454 PMC11687932

[50] Huang, J. & Ling, C. X. Using auc and accuracy in evaluating learning algorithms. *IEEE Trans. Knowl. Data Eng.* **17**, 299–310 (2005).

[51] Haixiang, G. et al. Learning from class-imbalanced data: Review of methods and applications. *Expert Syst. Appl.* **73**, 220–239 (2017).

[52] Xu, L. & Veeramachaneni, K. Synthesizing tabular data using generative adversarial networks (2018). ArXiv preprint, arxiv: 1811.11264v1.

[53] Demšar, J. Statistical comparisons of classifiers over multiple data sets. *J. Mach. Learn. Res.* **7**, 1–30 (2006).

[54] Saad, M. M., O’Reilly, R. & Rehmani, M. H. A survey on training challenges in generative adversarial networks for biomedical image analysis. *Artif. Intell. Rev.* **57**, 19 (2024).

[55] Ahmad, Z., Jaffri, Z. u. A., Chen, M. & Bao, S. Understanding gans: fundamentals, variants, training challenges, applications, and open problems. *Multimed. Tools Appl.* 1–77 (2024).

[56] Qin, X., Shi, H., Dong, X., Zhang, S. & Yuan, L. Improved generative adversarial imputation networks for missing data. *Appl. Intell.* **54**, 11068–11082 (2024).

